# Inter-Rater Reliability of Kampo Diagnosis for Chronic Diseases

**DOI:** 10.1089/acm.2020.0298

**Published:** 2021-07-12

**Authors:** Ayako Maeda-Minami, Tetsuhiro Yoshino, Yuko Horiba, Tomonori Nakamura, Kenji Watanabe

**Affiliations:** ^1^ Division of Pharmaceutical Care Sciences, Graduate School of Pharmacy, Keio University, Tokyo, Japan.; ^2^ Center for Kampo Medicine, Keio University School of Medicine, Tokyo, Japan.

**Keywords:** Kampo, pattern diagnosis, inter-rater reliability

## Abstract

This single-center observational study aimed to assess the inter-rater reliability (IRR) of Kampo medicine pattern diagnosis, which is modularized into three modules for chronic diseases, using 64 participants' information documents. The linearly weighted percentage of agreement and Gwet's agreement coefficient (AC) 2 for the deficiency–excess module, among three specialists, were 85.9% and 0.708, respectively. The unweighted percentage of agreement and Gwet's AC1 were 64.6% and 0.542 for the cold–heat, and 35.9% and 0.254 for the *qi*–blood–fluid modules, respectively. Our findings suggest that our modularization method may improve the IRR of Kampo medicine pattern diagnosis.

## Introduction

The 11th version of the International Classification of Diseases (ICD-11) was endorsed at the World Health Assembly in May, 2019^1^; it incorporated a chapter on traditional medicine, which included traditional Asian medicine. This ICD-11 chapter discussed two types of diagnoses: traditional medicine disorders and traditional medicine patterns.^1^ Traditional medicine patterns are defined as the comprehensive clinical presentations of a patient at a particular time point.^1^ The various traditional medicine patterns described in the chapter included principle-based (e.g., deficiency–excess and cold–heat) and body constituents (e.g., *qi*–blood–fluid) patterns.

As in conventional medicine, the validity and reliability of traditional medicine pattern diagnosis are also important, as they are essential components of ICD-11. Inter-rater reliability (IRR) is defined as the consistency of diagnostic assessments between two or more providers. However, the IRR of traditional Asian medicine diagnosis is low; this is likely due to the complexity and heterogeneity of the pattern diagnoses of traditional Asian medicine, lack of standardized terminology, and statistical problems.^2^ To overcome these challenges, a novel diagnostic system is necessary for a reliable and reproducible traditional medicine pattern diagnosis.

The probabilities of the frequency of traditional medicine pattern diagnosis are not uniform. Popplewell et al. suggested using Gwet's agreement coefficient (AC), which estimates agreement by chance. Using AC1 and AC2, one could accommodate the assessment of free marginal data and improve the evaluation of inter-rater agreement, compared with that obtained using Fleiss' Kappa, in Traditional Chinese Medicine (TCM) pattern diagnosis.^3^ AC1 is used for categorical data, while AC2 is used for ordinal data and can be used for weighted statistics.^3^

**FIG. 1.**
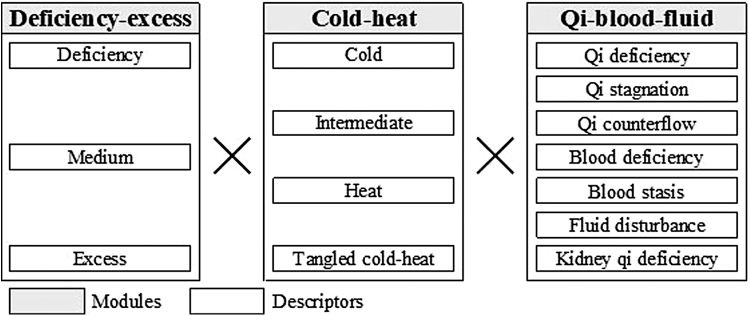
Figure diagnostic flow of Kampo pattern diagnosis for chronic diseases. Each patient is assessed across all three modules (deficiency–excess, cold–heat, and *qi*–blood–fluid) and their Kampo pattern diagnosis is determined by the multiplication of these modules.

Popplewell et al. also introduced the TCM diagnostic descriptor, which is a simplified version of the holistic TCM pattern diagnosis, for evaluating IRR.^4^ One possible option to further improve IRR is using a modularized approach.^5^ Indeed, Yakubo et al. proposed a modularized Kampo pattern for chronic diseases determined by three modules, namely, deficiency–excess (including three ordinal descriptors: excess, medium, and deficiency), cold–heat (four categorical descriptors: cold, intermediate, heat, and tangled cold–heat), and *qi*–blood–fluid (seven categorical descriptors: *qi* deficiency, *qi* stagnation, *qi* counter flow, blood deficiency, blood stasis, fluid disturbance, and kidney *qi* deficiency) (Fig. 1).^6^ Each patient is assessed across all three modules, and their Kampo pattern diagnosis is determined by the multiplication of these modules.^5^

This study aimed to calculate several indicators of IRR, including Gwet's AC for the Kampo medicine pattern diagnosis of chronic diseases, by following the modularization method proposed by Yakubo et al.^6^

The study design was approved by the appropriate institutional review board at Keio University (approval no.: 20100144).

## Materials and Methods

### Participants

This single-center cross-sectional study is a subset of a prospective cohort study, which enrolled all individuals who visited the Keio University Hospital's Kampo clinic from October 2014 to December 2017 and provided written informed consent for inclusion. We pooled 1286 participants who first visited the Kampo clinic during this period.

Sixty-four adult patients who had a typical *qi*–blood–fluid pattern were arbitrarily selected for this study, to achieve a uniform distribution in each *qi*–blood–fluid category. *Qi* counter flow is an exception since it is rarely diagnosed. Patient nos. 1–32 were selected from the outpatient clinic by doctor A, and patient nos. 33–64 by doctor B. From these 64 examinations, information documents were constructed for later analyses.

The physicians employed in this study had held active biomedical medical licenses for more than 10 years and were board certified by the Japan Society for Oriental Medicine, with 5 or more years of clinical experience.

### Information documents

In this study, the physicians were requested to evaluate the deficiency–excess, cold–heat, and *qi*–blood–fluid modules using only the information documents. These documents contained the results of a 140-item questionnaire that included 133 symptoms, chief complaints, systolic and diastolic blood pressure, age, sex, height, and weight.^7–9^ Patients' information such as their names, history of present illness, and physical examination findings (including pulse and abdominal examinations) was partially masked to the physicians.

### IRR; three doctors (doctors A, B, and C)

The inter-rater agreement of the traditional medicine pattern diagnosis with the information documents was evaluated between doctors A and B, as well as between those and a third doctor (C). All three doctors examined the information documents of all 64 patients, 3–5 years after the first consultation. The linearly weighted percentage of agreement was calculated for the ordinal descriptors in the deficiency–excess module, while the unweighted percentage of agreement was calculated for the categorical descriptors of the cold–heat and *qi*–blood–fluid modules. In addition, AC2 was calculated for the deficiency–excess module, and AC1 for the cold–heat and *qi*–blood–fluid models.

### Statistical analysis

R-software version 3.5.1 was used for all statistical analyses, and the irrCAC package^10^ was used to calculate the percentage of agreement, AC1, and AC2.

## Results

### Study participants

The most common biomedical diagnoses included cold hypersensitivity, climacteric syndrome, hypertension, and numbness of the limbs. Kampo diagnosis using the three modules at the patients' first consultation is given in Table 1. The distributions of deficiency–excess and cold–heat patterns in the 64 participants were similar to those in the 1286 pooled participants who first visited the Kampo clinic during the study period.

**Table 1.** Kampo Diagnosis Using Three Modules at First Consultation

Module	Descriptors	Doctor A	Doctor B	Total
		Patient nos. 1–32	Patient nos. 33–64	
Deficiency–excess	Deficiency	15	14	29
	Medium	12	7	19
	Excess	5	11	16
	Total	32	32	64
Cold–heat	Cold	16	17	33
	Intermediate	7	10	17
	Heat	3	4	7
	Tangled cold–heat	6	1	7
	Total	32	32	64
*Qi*–blood–fluid	*Qi* deficiency	5	5	10
	*Qi* stagnation	5	5	10
	*Qi* counterflow	2	3	5
	Blood deficiency	5	4	9
	Blood stasis	5	5	10
	Fluid disturbance	5	5	10
	Kidney *qi* deficiency	5	5	10
	Total	32	32	64

### IRR; three doctors (doctors A, B, and C)

The percentage of inter-rater agreement based on the questionnaire and AC2 for the deficiency–excess module were 85.9% and 0.708, respectively (Table 2). Moreover, the percentage of agreement and AC1 for the cold–heat module were 64.6% and 0.542, respectively, and those for the *qi*–blood–fluid module were 35.9% and 0.254, respectively.

**Table 2.** Inter-Rater Reliabilities Determined for Traditional Medicine Specialists, Based on the Findings from the Patient Questionnaire

	The number of categories	Percentage of agreement^a^	AC1/2^b^
Deficiency–excess	3	85.9	0.708
Cold–heat	4	64.6	0.542
*Qi*–blood–fluid	7	35.9	0.254

^a^ Percentage of agreement of deficiency–excess is linearly weighted, percentages of agreement of cold–heat and body constituents are unweighted.

^b^ AC2 is used for deficiency–excess because this is ordinal data and AC1 is used for cold–heat and body constituents because these are categorical data.

AC1, Gwet's agreement coefficient 1; AC2, Gwet's agreement coefficient 2.

## Discussion

We examined the IRR of Kampo medicine pattern diagnosis for chronic diseases using the modularization method. Our results suggest that modularization may improve the IRR assessment for each module of pattern diagnosis and help specialists reach an agreement. Combining these modules allows specialists to conclude a precise Kampo pattern diagnosis.

Inter-rater agreement of the *qi*–blood–fluid module was lower than that of the deficiency–excess and cold–heat modules. This finding may be attributed to the number of descriptors in each module. The deficiency–excess module has the fewest descriptors but the highest IRR, suggesting that modularization of traditional medicine pattern diagnosis could improve IRR. The IRR of the *qi*–blood–fluid module increased significantly when it was modularized into three categories (i.e., *qi*, blood, and fluid); however, these three categories exhibited too much granularity to be of use as final descriptors (data not shown). Yet, it may be possible to handle *qi*, blood, and fluid as three independent modules instead of combining them into a single module. This *post hoc* analysis also suggested that complexity and ambiguity of pattern diagnosis exist in the *qi*–blood–fluid module due to the “constitutional” similarity of several descriptors.

The relatively low IRR of the *qi*–blood–fluid module could also be affected by the module's heavy dependence on the physicians' physical examinations, including pulse and abdominal examinations, suggesting that assessments based solely on information documents were not enough. The IRRs of the deficiency–excess and *qi*–blood–fluid modules improved upon the addition of information regarding the physical examinations; however, those of the cold–heat module did not (data not shown).

We have reported a statistical model that predicted the specialist's deficiency–excess and cold–heat diagnosis. This machine-learning model showed a high prediction rate (70–90%) of specialists' diagnoses,^7–9^ and these diagnoses were predicted with the same information set used in this study. The predicted agreement rates for deficiency–excess and cold–heat modules using our model were comparable with the observed inter-rater agreement, and reproducibility between evaluation performed using information documents and diagnosis from patients (data not shown). Therefore, our prediction model for the deficiency–excess and cold–heat modules may help physicians, even during training, to reach a reasonable Kampo pattern diagnosis.

This study has several limitations that should be addressed by future studies. The Kampo specialists based their diagnosis on information reported in a patient questionnaire instead of an in-person examination. The three specialists who contributed to this study worked at the same center, which limits the generalizability of our study's results. Therefore, our results should be validated by multicenter studies. Finally, we did not perform direct comparisons between nonmodularized Kampo diagnosis and the current modularized diagnostic structure.

## Conclusions

In this study, we evaluated and confirmed IRR of Kampo pattern diagnosis for chronic diseases. The inter-rater agreements, based only on patient information, for the deficiency–excess and cold–heat modules were higher than those for the *qi*–blood–fluid module. These results suggest that the modularization of Kampo pattern diagnosis, with a smaller number of descriptors, could potentially improve IRR. Combining these modules allows specialists conclude a precise Kampo pattern diagnosis.
